# Health Taxes on Tobacco, Alcohol, Food and Drinks in Low- and Middle-Income Countries: A Scoping Review of Policy Content, Actors, Process and Context

**DOI:** 10.34172/ijhpm.2020.170

**Published:** 2020-09-06

**Authors:** Lana M. Elliott, Sarah L. Dalglish, Stephanie M. Topp

**Affiliations:** ^1^College of Public Health, Medical and Veterinary Sciences, James Cook University, Townsville, QLD, Australia.; ^2^School of Public Health & Social Work, Queensland University of Technology, Brisbane, QLD, Australia.; ^3^Department of International Health, Johns Hopkins School of Public Health, Baltimore, MD, USA.; ^4^Institute for Global Health, University College London, London, UK.; ^5^Nossal Institute for Global Health, University of Melbourne, Melbourne, Australia.

**Keywords:** Non-communicable Diseases, NCDs, Fiscal, Tax, Policy, LMICs

## Abstract

**Background: **Taxation of tobacco, food, alcohol and other beverages has gained renewed attention in responding to non-communicable diseases (NCDs). While largely built on evidence from high-income countries (HICs), the projected economic and health benefits of these measures have increased calls for their use in price-sensitive low- and middle-income countries (LMICs). However, uptake has been sporadic and there remains little research on why and how LMICs utilise fiscal measures in response to NCDs.

**Methods: **This scoping review analyses factors influencing the design and implementation of health-related fiscal measures in LMICs. Utilising Arksey and O’Malley’s scoping review methodology and Walt and Gilson’s policy triangle, we considered the contextual, procedural, content and stakeholder-related factors that influenced measures.

**Results:** We identified 75 papers focussing on health-related fiscal measures, with 47 (63%) focused on tobacco, 5 on alcohol, 6 on soft drink and 4 studies on food-related fiscal regulation. Thirteen papers analysed multiple measures and most papers (n = 66, 88%) were less than a decade old. Key factors enabling the design and implementation of measures included localised health and economic evidence, policy championing, inter-ministerial support, and global or regional momentum. Impeding factors encompassed negative framing and retaliation by industry, vested interests and governmental policy disjuncture. Aligning with theoretic insights from the policy triangle, findings consistently demonstrated that the interplay between factors – rather than the presence or absence of particular factors – has the most profound impact on policy implementation.

**Conclusion: **Given the growing urgency to address NCDs in LMICs, this review highlights the need for recognition and rigorous exploration of political economy factors influencing the design and implementation of fiscal measures. Broader LMIC-specific empirical research is needed to overcome an implication noted in much of the literature: that mechanisms used to enact tobacco taxation are universally applicable to measures targeting foods, alcohol and other beverages.

## Introduction

 Non-communicable diseases (NCDs) are now the leading cause of death and disability, resulting in more than 41 million deaths annually and accounting for 71% of global mortality.^1-3^ Of the total NCD burden, 80% is attributed to cancer, diabetes, cardiovascular disease and chronic obstructive pulmonary disease, conditions that are largely preventable and driven by the risk factors of smoking, alcohol consumption, inadequate physical activity and poor diet.^4^ The global escalation in NCDs represents a threat to the health of populations, stability and responsiveness of health systems and the viability of national economic progression.^5^ It is for these reasons that NCDs have been identified by the World Economic Forum as one of the greatest global threats to economic development.^6^

 The multisectoral nature of NCD determinants requires intervention beyond the health sector and demands policy consensus across diverse stakeholders.^7^ This multisectoral approach recognises that many decisions affecting the prevalence and impact of NCDs are determined by national and international policies related to trade, agriculture, urban planning and finance-interested parties in boardrooms of national and multinational corporations.^7-9^ As such, global recommendations for addressing NCDs increasingly reference and seek to address the underlying social and commercial determinants of health.^10-12^ Global recommendations focusing on supporting population behaviour change and minimising the impact of health-harming practices by corporations through enhanced regulation are bundled into packages such as the World Health Organization (WHO) NCD Best Buys.^6,13^

 The use of fiscal measures to limit the impact of health-harming commodities, such as alcohol and tobacco, is not new.^14^ However, this policy space has gained additional attention in the last decade given the escalation of NCDs.^15,16^ Fiscal measures targeting tobacco and alcohol are now present in 161 and 156 countries respectively, having gained traction from the Framework Convention of Tobacco Control (FCTC),^17^ the Global Strategy to Reduce the Harmful use of Alcohol^18^ and the NCD Best Buys.^6,19,20^ Drawing on tobacco and alcohol taxation successes, measures have also been adapted and enhanced for use on a broader range of harmful commodities.^21^ Fiscal measures targeting sugar sweetened beverages, ultra-processed and energy-dense foods are now present in more than 45 countries and local jurisdictions globally.^22-24^

 Research analysing the economic implications of fiscal measures is vast. However, the health literature in this space is concentrated on predictive forecasts and, to a lesser but growing extent, empirical ex post studies focused on high-income countries (HICs).^25^ Positive findings identifying economic and public health gains from fiscal measures hence derive almost exclusively from HIC-specific data, yet these measures are widely recommended for use in the more price sensitive markets of low- and middle-income countries (LMICs).^25-27^ With different political and economic contexts, the assumption that the political appetite, policy process and potential impact in LMICs will mirror HIC case studies is questionable and remains unsubstantiated by sufficient evidence.^27-29^ For example, the introduction of sugar sweetened beverage taxation in 31% of HICs compared to just 13% of LMICs^23^ questions whether measures can be successfully implemented in the distinctive political and economics landscapes of LMICs.

 Although still in its infancy, there is growing research interest in fiscal measures in LMICs. Studies by Nakhimosky et al,^30^ Sassi et al,^31^ Sornpaisarn et al^32^ have begun to demonstrate the economic impact of fiscal measures in LMICs specifically. While Bump and Reich’s 2013 analysis of tobacco-specific fiscal measures provided one of the earliest political economy examples.^33^ This paper highlighted the important but under-researched articulation of the influence of political and economic dynamics on policy adoption. More recent work includes Wright and colleagues’ study reviewing global harmful commodity tax measures^25^; Hagenaars and colleagues’ work on policy content and policy context of energy dense food and sugar-sweetened beverage taxation in 13 (majority HIC) case studies^24^; and Bridge and colleagues’ overview of LMIC’s experiences in implementing soft drink specific fiscal measures.^34^ Building on these examples, but utilising a systematic scoping review and focussing on LMIC specifically, this paper aims to: (1) map evidence relating to policy content, stakeholder-related, procedural and contextual factor that shape harmful commodities focused fiscal measures; (2) identify points of convergence and divergence across the LMIC literature; and (3) compare LMIC specific findings with fiscal measures focused literature from HICs.

## Methods

 This study utilised scoping review methods developed by Arksey and O’Malley^35^ to identify, map and highlight potential gaps in LMIC policy process-relevant fiscal measures research. Detail on the application of the 5-stage framework, encompassing: (1) question identification, (2) study identification, (3) study selection, (4) data charting, and (5) collation and synthesis, is outlined below.

 The research question emerged through iterative exploration of the broad themes of ‘fiscal policy’ and ‘NCDs.’ Acknowledging the breadth of this domain, source and study selection sought to balance comprehensiveness with feasibility.^35^

###  Database Search Strategy

 Four databases (PubMed, Embase, ProQuest, and Scopus) were searched using the key terms ‘health policy,’ ‘regulatory,’ ‘LMICs,’ ‘NCDs,’ ‘harmful commodities’ and their derivatives separated by the Boolean operator ‘AND’ for all except NCDs and harmful commodities where ‘OR’ was utilised to more comprehensively canvas relevant papers. We used broad search terms to capture additional fiscal measures content in research discussing policy responses to NCDs and their determinants more broadly. The full search strategy can be found in Supplementary file 1.

 Database searches returned 5047 papers, yielding 4669 original English works once duplicates were removed (March 2019). As represented in Figure, articles were then excluded sequentially by the first author in consultation with the third/senior author based on title, abstract and full text. Inclusion criteria centred on papers demonstrating a focus on LMICs and policy process while also exhibiting an explicit link between health and fiscal measures. Application of this criteria led to the exclusion of 3660 papers based on titles. More specific criteria were subsequently applied to abstract and full-text screening to identify papers that provided explicit accounts of agenda setting, design and implementation of fiscal measures. These criteria excluded non-empirical works and prevalence, predictive, experimental, and outcome/impact-focused studies. A summary of inclusion/exclusion criteria can be found in Supplementary file 2. Abstract screening of 1009 works excluded 914 papers with many lacking empirical basis (n = 211) or a specific focus on fiscal measures (n = 296). Full text screening of the remaining 95 papers excluded an additional 44 lacking underpinning policy process focus (n = 23). This left a final set of 51 peer-reviewed research papers and book chapters. References lists of included papers and excluded papers of note were searched by hand resulting in the inclusion of 12 additional resources.

**Figure:**
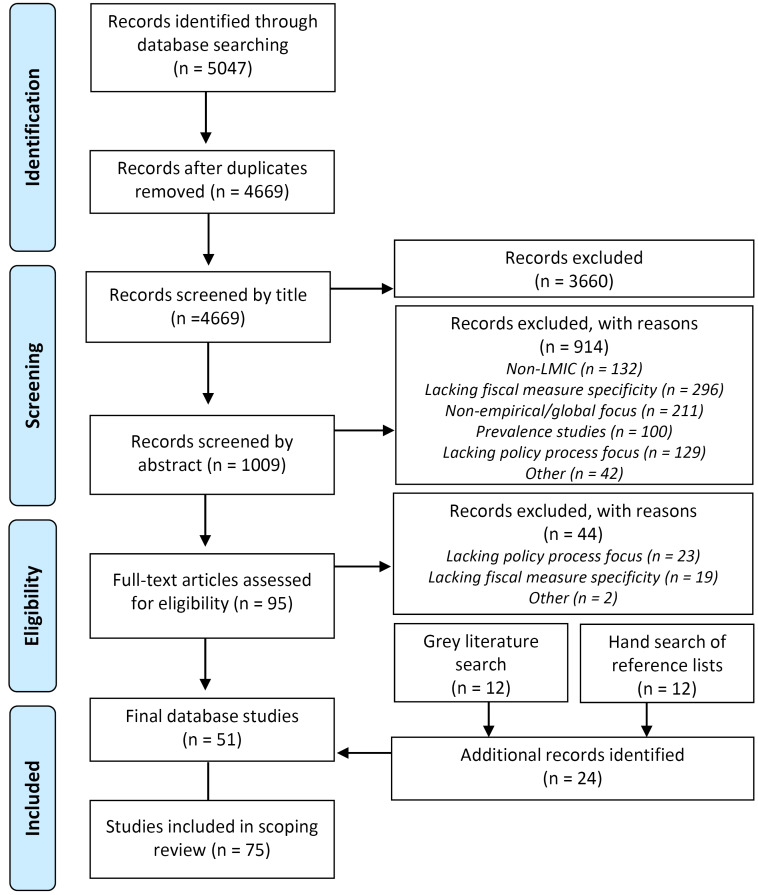
Selection Procedure of Included Studies.

###  Grey Literature Search Strategy

 Grey literature was also sourced through selected agencies: WHO, World Bank, and International Monetary Fund (IMF). The grey literature search strategy applied the same key words and inclusion/exclusion parameters as those used in the academic database search and was undertaken by the first author in consultation with the third author. Publication repositories and search functions on each website were searched first. However, acknowledging the often-limited functionality of these mechanisms, Google domain searches were also conducted. Most of the reviewed reports and publications were excluded given their prospective focus on the projected benefits of measures. A total of 12 grey literature publications were included.

 A final set of 75 papers, reports and book chapters were obtained for analysis through the combined database, grey literature and hand search strategies.

###  Data Synthesis and Analysis

 We conducted data charting, collation and synthesis using an iterative approach and following Ritchie and Spencer’s^36^qualitative data analysis framework. Data familiarisation was undertaken by the first author by generating a list of key ideas covered by the 75 included papers. These ideas formed the basis for an initial codebook workshopped by the first and third author, found in Supplementary file 3. A first round of coding was conducted using NVivo 12.^37^ The first and third author reviewed these codes and considered the data in light of various theoretical frameworks. Walt and Gilson’s^38^ policy triangle was selected to further refine analysis. The policy triangle is derived from LMIC-specific policy analysis and has been used in similar studies, including those by Hagenaars et al^24^ and Downs et al.^39^ While acknowledging inevitable overlap, blurred boundaries and dynamic interaction between the domains of policy content, actors, process and context, it was selected given its ability to support the identification of diverse factors and dynamics influencing the design and implementation of fiscal measures.

 Recoding was undertaken utilising a refined codebook with codes pre-grouped corresponding with each policy triangle domain and, in the case of contextual factors, further refined by Leitcher’s^40^ framework of situations, structural, cultural and international/exogenous factors.^38^ Coding summaries were produced and used to visually map findings; these maps supported interpretation and write-up, using both deductive and inductive reasoning. The first author undertook preliminary synthesis of thematic areas, followed by consultation and critical evaluation by the second and third authors to provide additional depth of analysis. Table 1 summarises the 4 domains of the policy triangle and respective themes and subthemes that emerged during analysis.

**Table 1:** Description of the Policy Triangle Domains and Identification of Themes^38,40^

**Domains**	**Description of Domains**	**Identified Themes **
Content	The technical content or prescriptive detail included in policy documents.	• Earmarking
		• Taxation scope, rate and tiered structure
Actors	The individuals, groups and organisations who interact with and influence policy.	• The influence of industry
		• Policy champions
		• Civil society engagement
		• Multilateral actors
Process	The actions that influence how issues are recognised and how policies are designed, negotiated, communicated, implemented and evaluated.	• Framing
		• Evidence
		• Inter-ministerial policy dynamics
		• Implementation
Context	The political, social, cultural, economic and international bounds in which actors work and policies are devised.	• Situational factors
		• Structural factors
		• Cultural factors
		• International/exogenous factors

## Results

 Results indicate that the study of policy processes of health-related fiscal measures in LMICs is an emerging field. The earliest included paper in this study was published in 1998 while most papers (n = 64) were published between 2009 and early 2019. Of the 75 papers identified, 47 studies (63%) focused on tobacco-related fiscal measures. Alcohol was the focus of 5 studies, while soft drink and food-related fiscal regulation were the focus of 6 and 4 papers, respectively. Thirteen papers sought to analyse more than one commodity-related fiscal measure. Utilising the World Bank 2019 categorization of income status,^41^ low-income countries were the focus of just 2 papers. Nineteen papers (25%) focused on fiscal measures initiated in lower-middle income countries while 28 (37%) analysed upper-middle income countries. The remaining 26 papers (35%) made comparisons across multiple countries with varying socioeconomic statuses. Geographically, Asia (n = 21) and Africa (n = 20) were the predominant regions of focus. South and Central America (n = 10) and the Pacific (n = 6) also featured prominently. While just 2 papers focused on Europe and one paper on the Middle East, this needs to be considered in light of the study’s LMIC-focus. Fifteen papers utilised cross-regional comparisons. A table incorporating the main attributes of each paper and their identification of themes linked to Walt and Gilson’s^38^ policy triangle can be found in Supplementary file 4.

###  Policy Content

 The prescriptive detail and technical content outlined in policy documents is integral to conveying and achieving policy objectives. Two major themes were identified in the analysis of policy content: earmarking of revenue and tiered taxation measures.

####  Earmarking

 Earmarking (ie, assigning government revenue to a specific purpose)^42^ was a commonly discussed policy mechanism in the reviewed literature. Administered through formal legal process in some instances and more symbolic forms of policy in others, the earmarking of funds garnered support for fiscal measures in some cases while fuelling inter-ministerial conflicts in others.

 The benefits of earmarking to fiscal measure adoption were examined by 12 papers spanning the Pacific, Asia, South America, and Africa.^15,42-52^ Papers identified varied applications of dedicated revenue including additional non-specific budgetary allocations to health^51^; the development of health promotion funds or foundations^15,43,47,50-52^; subsidies for healthier alternatives^15,50^; tagged funding for organisational units^45,49^; and equity-based measures dedicating funds to universal health coverage or reparations for affected farmers and communities.^42-44,46,48^ While linked to diverse goals, earmarked funds can garner public and political support for fiscal measures by acting as a traceable policy output; overcoming a common retort that measures purely focus on revenue generation.^44,47,53^ In contrast however, a number of papers also identified earmarking to circumvent general public financial management; fuelling inter-ministerial conflicts and stalling or mitigating fiscal measures.^42,54-57^ Given the overlap between the domains of policy content and policy process, deliberative decision-making surrounding earmarking will be further discussed in the policy process domain.

####  Taxation Scope, Rate and Tiered Structures

 Most fiscal measures in LMICs target single commodities with rigid parameters. Broader measures in some Small Island Developing States and Mexico are rare exceptions.^51,58-60^ This well-defined focus on a single commodity may permit consumers to substitute purchases with similarly unhealthy products, potentially undermining the health impact of the measure.^24,51^ However, as Hagenaars et al^24^ argue, a well-defined scope strengthens governments’ defence of measures in the face of corporate, community or international trade disputes. The scope of implemented measures may thus reflect a trade-off between public and political acceptability of the measure and effectiveness insofar as its impact on population health.

 Literature also outlined diversity in the type of taxation prescribed. Taxation generally encompassed combinations of specific, ad valorem (a proportion of the estimated product value), value added and import taxations. Taxation rates also varied between and within commodities from as little as US$0.05 per packet of cigarette in Costa Rica,^61^ to as much as a 300% import levy on turkey tails in Samoa.^50,62^ Variability across measures and contexts indicates that fiscal measures are often designed to reflect local needs even if the catalyst for their use was external, such as ‘global best practice’ guidelines or the experience of other nations.

 Governance and administrative benefits of simplified and uniform taxation measures were explicated by some papers,^48,63,64^ yet tiered structures, with rates based on production capacity or concentration of particular ingredients, were also common.^55,59,65-68^ Tiered taxations are promoted by a growing body of literature, including Baker et al^53^ and Roubal^55^ in relation to sweetened drinks, as a means of incentivising product reformulation.^46,48,51^ However, other sources suggest that tiered structures associated with some commodities can reflect industry involvement in weakening measures; ensuring that they are geared towards competitors or smaller fragments of the market.^46,63,66^ This has been the case with tobacco in Indonesia, where tiered measures have embedded conditions favourable to industry.^65-68^ As alluded to by Williams^64^ and Kaiser et al,^48^ if insufficiently monitored, tiered measures may also complicate administration and risk diminishing the health impact of fiscal measures.

###  Policy Actors

 Policy actors are individuals or groups who, formally or informally, are involved in the policy process.^69^ However, who is considered a policy actor, the power they wield and how interests are negotiated in formulating policy, depends on context and process.

####  The Influence of Industry 

 Industry influence was the most commonly identified theme across the literature, covered by 88% (n = 66) of sources. Spanning conceptualisation, design, implementation and sustainability of fiscal measures, industry influence encompassed a range of tactics, criticisms and defences utilised by national and international corporations whose products were subject to proposed or introduced taxations.

 The literature describes industries’ use of pre-emptive action to stave off regulatory measures. Intentional framing of industry activity as a significant source of LMIC employment and contributor to gross domestic product was a common tactic used to influence public and political opinion of fiscal measures.^47-49,53,60,64,68,70-83^ Framing was often coupled with the projection of negative ramifications for economically-vulnerable primary producers.^48,57,65,71,73,78,81,82,84,85^ This tactic was explicitly used by the International Tobacco Growers Association in response to mounting pressure for tobacco taxation in sub-Saharan Africa.^82^ Framing of economic flow-on effects permits industry advocates to paint fiscal measures as not only a threat to their bottom line but also to primary producers, employees and national economic prosperity. By extension, Mialon et al^86^ and Chavez^44^ also highlight the industry threat of offshoring; claiming fiscal measures to undermine the economic viability of local production. Such threats strike at LMICs’ economic interests and may ignite or fuel inter-ministerial conflicts.^70,75,87,88^ In some cases, threats have been compounded with real or perceived threats of retaliative trade action.^33,50,59,73,86,89,90^

 Industry advocates also commonly highlight perceived unintended consequences of fiscal measures. Commonly identified consequences include cigarette smuggling^33,42,45,48,54,56,57,61,67,70,73,81,90-97^; a shift towards informal home-brewed alcohol^48^; and food and beverage unaffordability, nutrient deficiency and dehydration.^64,91^ Findings from Abedian et al and Coriakula et al^53,98^ suggest strategic links to the media, or indeed media ownership itself, to perpetuate this framing in the public sphere. Corporate social responsibility initiatives are also a common industry tactic to seek favourable public positioning.^64,68,77,90,99^ These covert industry tactics are also coupled with more overt mechanisms for mitigating the adoption of measures. Common techniques include fostering and embracing ties to the political and economic elite^46,75,77,81,84,86,87,99,100^; funding or supporting political parties^86^; and establishing lobbying and front groups prone to deception, disputing evidence and directly interfering in policy process.^15,42,46,47,49,51,53,55,56,59,60,63,65,66,68,71,73,79,85,86,94,96-99,101-104^ Industry interference often results in stalled or diluted fiscal mechanisms; undermining the strength and sustainability of measures.^45,49,51,53,61,66,68,73,79,81,82,84,87,89,93,94,97,99,102,103,105,106^ Lack of confidence in the relative advantage of different policy options is also commonly exploited by industry advocates as an opportunity to propose alternative, self-regulatory measures.^55,59,67,86^

 Following the implementation of fiscal measures, corporate retaliation is sustained. Responses often align with previous pre-emptive action and seek to minimise impact on profits. Responses include offshoring to more favourable economic conditions, as noted by Holden & Lee in Central America^100^; restructuring manufacturing or labelling to exploit tiered or poorly constructed measures^65,101^; pursuing trade or legal recourse^53^; adjusting price to maintain market share or profitability^92,95,107^; and paying further lip service to, if not actively engaging in smuggling as identified by Van Walbeek.^56^

####  Policy Champions

 Forty-six papers (61%) detailed the influence of political commitment and leadership on the design and implementation of fiscal measures. Studies highlighted that effective implementation is contingent upon sustained endorsement and policy championing by executive levels of government.^46,48,49,52,54,56,60,62,63,68,79-82,84,87,97,98,101,108-111^ Most commonly, this consisted of public advocacy for measures by Presidents, Prime Ministers and national Ministers of Health. However, motivation underpinning elite support varied and included electoral commitments to tax or health reform^48,60,80^; changing political climates, as was the case in post-Apartheid South Africa^56,71,81,82,84,97^ and the fall of the New Order in Indonesia^68^; and personal convictions such as Prime Minister Erdogan in Turkey,^109^ President Yar’Adua in Nigeria^110^ and President Batlle in Uruguay.^108^ Sources indicate that the efficacy of state actor policy championing improved when advocacy was evidence-informed and recognised the harms associated with particular commodities.^42,48,49,79,101^ Mapa-Tassoa et al^78^ identified that ad-hoc and reactive instances of policy support by political elites were more likely to result in under-resourced, conflicting and piecemeal policy responses. Similar findings were echoed by Vateesatoket,^52^ who emphasised senior health experts to have a more sustained commitment to measures than career politicians.

 Papers that analysed unsuccessful measures found that these measures were commonly undermined by a lack of sustained political commitment.^65,74,75,78,92,96,104,105,112^ Political ambivalence or lack of support at elite levels was often driven by industry framing of the perceived economic and political risks of taxation.^42,65,74,85,93^ As identified by Barraclough and Morrow^72^, the combination of industry framing and political ambivalence commonly resulted in political elites compromising their regulatory responsibilities in favour of potential political and economic gains. The scarcity of financial and human resources in the health sector also resulted in the prioritisation of responses to communicable diseases at the expense of actions to address NCDs.^49,76,87,89,104,105,112^ Ferreira-Borges et al^89^ further highlight that insufficient prioritisation of NCDs can be reinforced by the funding priorities of international donors.

####  Civil Society Engagement

 Civil society engagement was identified as integral to successful fiscal measures in 35 papers (47%). Civil society groups included local, national and international research agencies and academics, special interest communities, and non-government organisations (NGOs) whose interests were strategically aligned.^15,42,48,52,61,82^ Spanning diverse contexts and commodities, these bodies often shaped public and political agendas,^33,76,87^ disseminated policy-relevant research,^61,113^ countered industry claims,^73,82,110^ and held governments to account.^42,48,52,55,56,60^ As demonstrated by lobbying from health professionals, researchers and civil society activists during the introduction of tobacco-related measures in South Africa,^71,81,84^ effective civil society groups often had high visibility and links to media and government.^53,84,103,109,111^ As elucidated by Kaiser et al,^48^ such groups often work across government and civil society to construct robust measures which bridge tensions and are less susceptible to hijacking by detractors. However, Barraclough and Morrow,^72^ Higashi et al^74^ and Mapa-Tassoa et al,^78^ found that the power of civil institutions diminished when targeted commodities had high public acceptance or where state ownership or sponsorship stifled the proposition of change.

####  Multilateral Actors

 Direct support by multilateral agencies, NGOs and philanthropic trusts was identified to expedite the design and implementation of fiscal measures. While the WHO was held in high regard for their technical advice,^45,55,60,63,98^ sources also highlighted multisectoral consensus building to derive from engagement with the Food and Agriculture Organization (FAO), IMF, the World Bank, and other national and international NGOs and philanthropic trusts.^53,61,62,68,71,73,104,113^ Locally-based offices of multilateral agencies, seed organisations and links between multi-lateral actors and local organisations sometimes blurred the boundaries between multilateral and civil society action. Financing and the supplementation of national economic, technical and legal capacities were the predominant forms of support, with each noted to be beneficial in overcoming common LMIC capacity constraints.^53,60,61^

###  Process

 Literature provided insights into 4 domains, also commonly identified in policy process theory^114-119^: the strategic portrayal of policy problems and fiscal solutions – often called ‘framing’^120^, the forms and use of evidence, inter-ministerial policy dynamics and policy implementation.

####  Framing

 Literature identified three central frames: pro-health, pro-economic and (often industry-induced) scepticism of fiscal measures.^24,50-52,55,64,81^ As argued by Kaiser et al,^48^ these three unidimensional frames foster policy coalitions capable of elevating issues onto the political agenda. This was evidenced in links between multinational corporations, small business and unions in response to potential employment repercussions of fiscal measures^55,65,83^; and in coalitions formed between health professionals and families with claims of industry putting profits before health.^52,53,92^

 The design and implementation phases of fiscal measures sometimes saw the emergence of nuanced frames capable of bridging more disparate interests.^53^ Onagan et al^63^ observed that measures framed with exclusive health or revenue objectives gained less inter-ministerial traction than those proposing dual objectives. The ‘win-win’ phraseology,^121-123^ often used to account for the projected health and economic benefits of fiscal health policy, is hence a particularly useful and well used frame for uniting otherwise disparate coalitions around mutually-beneficial goals.^43,48,50,51,55,60,63^

 Alignment with the global health agenda, insofar as ratification of FCTC^67,99,110,112^ and revenue generation to support universal health coverage,^42,46,48^ were also beneficial frames capable of driving implementation. While evidence-based frames, which drew on diverse projections of impacts, were most likely to mobilise ideologically and politically dissimilar audiences; giving political and social traction to measures.^48,53,63,71,73,85,98,104^ This dynamic is best demonstrated by the Philippines’ and Thailand’s additional revenue investments in health, which led to policy backing by health-interested parties rather than those solely interested in economic benefits.^48,52,63,124^

####  Evidence

 The role of local, regional, global and industry-endorsed evidence was discussed in 45 papers (60%). As a whole, the literature emphasised that fiscal measures gained traction from diverse types of country-specific evidence.^47,52,55,56,63,74,81,84,87,91^ As articulated by Higashi et al^47^ and Hamann et al^113^ however, the appetite for context-specific evidence is not always matched by research availability, affordability or the capacity of LMIC governments and research institutions. Paucity of local evidence often forced governments to choose between stalling policy to await relevant evidence or, acknowledging momentum and proceeding with suboptimal information.^49,52,74,79,85,113^ Diffusion of regional or global evidence was beneficial in progressing fiscal measures and supported by pro-policy political elites and global health bodies.^15,55,60^ The use of more generalised data however was often criticised by industry and those opposing measures.^24,52,86^ In such instances, the availability of local albeit poorer quality evidence, often favouring or funded by industry, was enough to seed doubt amongst policy-makers, forcing compromise or delay.^66,73,79^

 Evidence was predominantly used to justify measures by highlighting the magnitude of health burden or economic deficit.^15,24,42,60,82,91,101,109,111^ Commonly identified evidence included consumption patterns, disease burdens, price elasticity and revenue or health projections associated with policy initiation.^15,24,42,53,63,81,82,91,109,111^ Such evidence was beneficial in countering industry claims,^42,56,61^ supporting technical provisions,^55^ and mobilising and sustaining civil society groups and messaging.^76,81^ However across the literature, evidence was almost exclusively used to justify implementation but not inform the design of policy per se.^80^

 Further, while few papers explicitly acknowledged the central role of political process on the design and implementation of measures, a handful of papers did recognise what is best surmised by Chantornvong et al^105^: that policy success depends as much on forms of “political evidence” as it does the application of health and economic specific knowledge.^33,48,51,73,80,97,105^ However, even amongst papers where political considerations were acknowledged, there remained limited empiric analysis and continued reliance on commentary, with the noteworthy exception of Kaiser and colleagues’^48^ political economy insights from the Philippines. As Sanni et al^97^ attest, technical evidence alone is insufficient to address politically contentious issues. Given the potential for political economy considerations to contribute positively to policy adoption, the deficit of evidence pertaining to political process and power dynamics is likely to be a missed opportunity to improve the design and implementation of measures.^33,48,51,73,74,80,84,97,105^

####  Inter-Ministerial Policy Dynamics 

 The multisectoral nature of fiscal measures requires policy development to bring together an extensive stakeholder base with diverse and, at times, conflicting priorities. At the intersection of health, trade, financial and agricultural interests, fiscal measures commonly fuel conflicts over policy jurisdictions.^44,47,53,56,70,71,73,76,91,97^ As outlined by Abediyi et al^70^ while dependent on framing and government objectives, cross-ministerial negotiations commonly stall or mitigate adoption. Pitso et al^49^ and Bump et al^73^ assert that, particularly in LMICs, inter-ministerial power asymmetries often favour financial and trade interests over health. If health measures are designed as part of broader fiscal reform, as was the case in Mexico^60^ and Tonga’s second and third iterations,^58^ this can accelerate implementation and permit governments to promote dual health and economic benefits.^42,91^ By contrast, when public sector and political officials perceive fiscal measures to challenge trade or economic interests, policy is often thwarted or enacted with health trade-offs.^48^ Protection of state-owned commodity producers and, as Abedian et al^54^ identify, the avoidance of earmarking revenue, are identified as 2 common trade-offs.^72,74^ In particular, earmarking is likely to draw opposition from national finance departments where, Van Walbeek^56^ argues, it may be perceived as economically inefficient and a violation of their role as gatekeepers of public financing. A push for earmarking, particularly when there is a distinct power imbalance between health and finance departments, has been identified by some sources to risk destabilising necessary multisectoral support for adoption of such measures.^42,54-57^

 Despite the frequency of inter-ministerial conflicts in this space, papers also identified instances of inter-ministerial collaboration and its positive implications on the design and implementation of fiscal measures.^15,43,48,52,58,63,71,79,80,91,101,104,108,111^ Often linked to strategically developed multisectoral coalitions, the literature emphasised the importance of coordinating policy responses,^76,89^ and striving for genuine integration.^79,91,111^ Acknowledging the diverse interests of stakeholders, Coriakula et al^101^ highlight the effectiveness of inter-sectoral committee mechanisms in ensuring collaborative and iterative policy development capable of harmonising and overcoming potential policy conflicts.

####  Implementation

 Notwithstanding the automated nature of taxation compared to other policy instruments,^125^ included literature outlined poor resourcing, vested interests and the lack of timely monitoring as implementation challenges prone to undermining the success of fiscal measures. Insufficient supportive resourcing was identified as significantly impacting implementation.^50,76,79,82,87,110,124^ As noted by Barber and Ahsan,^83^ this was particularly apparent in administering tiered taxation measures prone to industry manipulation and insufficient oversight. Producers and suppliers were also found to engage in a range of tactics to undermine the effectiveness of implemented measures, including relabelling,^50,101^ absorbing taxation to maintain market share,^51,83^ supporting the importation of cheaper alternatives,^51,83^ and price over-adjustment to sustain profits.^67^ These tactics were also commonly aided by concurrent political disunity. Competing government interests and complex policy sign-off processes were identified as factors responsible for delaying the implementation of measures.^50,59,84,90,92,97,98,103,104,110^ Similar challenges, coupled with ill-defined success, insufficient resourcing and complex causal pathways were also identified as limiting effective policy monitoring and evaluation.^24,42,51,83^ Thus, frequently, little is known about the intended or unintended consequences of measures, as required to inform future adjustments.

###  Context

 Policy context analysis recognises that policy actors and processes exist within a context that ultimately influences the design and outcome of reform. Leichter’s^40^distinction between interlinked situations, structural, cultural and exogenous/international factors is a useful heuristic for consideration of contextual factors and their influence on policy.

####  Situational Factors

 Situational factors encompass focusing events or diffuse recognition of issues which elicit a policy response.^40^ The majority of papers linked fiscal measures to diffuse recognition, while some included papers (n = 23) explicitly delved into the situational “tipping point” responsible for the ideation of measures. From a health standpoint, lobbying from civil society was identified as beneficial in elevating the health burden and potential solution represented by fiscal measures with respect to the public, media and political agendas.^80,82,97,98,110^ Comparative analysis of tobacco measures by Sanni et al^97^ highlighted how the emergence of context-specific, localised evidence justified expedited implementation in South Africa, while similar measures stalled in Togo without such localised evidence. Far more frequently, however, demand for government revenue expansion was used to justify the design and implementation of measures.^24,46,48,51,53,60,78,91,92,100,109,126^ This was commonly linked to shrinking revenue from exports,^91^ increased demand for public expenditure,^48,91^ the ramifications of trade liberalisation on import tariffs^24,51,53,78,100^ and state-ownership or monopolies.^46,92,109^ While revenue generation motives were effective in garnering initial support from economically-interested parties, as articulated by Kaiser et al,^48^ the design of measures necessitates the incorporation of a broader perspective, such as good governance or pro-poor sentiments, to sustain necessary backing from stakeholders.

 While not sparking issue recognition per se, election periods and political change-overs commonly enabled the implementation of fiscal measures.^56,61,68,81,82,97,101,109,110^ As a legacy of exiting leaders or the fulfilment of election promises by incumbents, fiscal measures that had already gained recognition on the policy agenda, were often expedited during leadership change-over.^56,61,68,81,82,97,101,109,110^

####  Structural Factors

 Despite the heterogeneity of LMIC contexts, papers commonly outlined demographic and epidemiological challenges; capacity constraints and institutional norms as interlinked structural influences on fiscal measures in LMICs.

 The economic development status of countries affected political and public receptiveness to proposed fiscal policy solutions to NCDs. For example, the double burden of NCDs and communicable diseases^127^ experienced by many LMICs was, as previously articulated, detrimental to NCD prioritisation.^49,76,87,89,104,105,112^ However, various sources found that globalisation, urbanisation and climate change-induced food insecurity had amplified the burden of NCDs; increasing demand for effective policy responses.^24,50,58,59,62^ Population growth and increasing wealth have also made LMICs a more favourable market for the sellers of harmful commodities.^48,70-72,82,83,90,98,99,103,104,124^

 Development-associated capacity constraints also challenge the design and implementation of fiscal measures. These include financial constraints,^73,76,78,79,82,87,92,104,110,124^ insufficient human resource and technical capacities to effectively negotiate trade deals,^45,50,59,90,103^ and sufficient resources to monitor and adjust fiscal measures where necessary.^15,47,55,68,71,72,74,81,84,85,97,112^

 The political structure and prevailing institutional norms also govern how states engage in the design and implementation of fiscal measures. Literature identified state-ownership^15,47,64,68,72,74,75,104^; culture of industry engagement and acceptance of their framing of issues^59,66,77,86,93^; inter-ministerial hierarchies^44,47,73,82^; policy space afforded to civil society^53,68,72,78,104^; and, on one cited occasion, neoliberalism,^98^ as norms that shaped measures. The impact of these norms is best exemplified by the contrasting successes in tobacco-related measures in post-Apartheid South Africa^56,81^ and challenges posed by state-ownership and industry influence on measures in Indonesia,^68^ China,^75^ and Vietnam.^47,74,104^

####  Cultural Factors

 Cultural factors, inclusive of history, religious sentiments and social structures have had a distinct influence on the design and implementation of fiscal measures.^40^ Commonly linked to consumption trends and cultural significance^65,68,89,98^ and historic links between government and local commodity producers^49,54,56,64,66,71,72,81,82,85,94,97,98^ cultural factors were often identified as key stalling points in the adoption of fiscal measures. As identified by Barraclough and Morrow^72^ and Ferreira-Borges et al,^89^ these factors were sometimes exploited by industry and those sympathetic to industry to delay or seed doubts around measures’ viability. By contrast, Achadi et al^65^ and Hoe et al^109^ found that religious objections to tobacco were beneficial in fostering a favourable political climate for fiscal measures in Indonesia and Turkey respectively. Growing nationalism also supported fiscal measures in post-apartheid South Africa^56,71,81,84^ and in response to pressure from the General Agreement on Tariffs and Trade for tobacco market liberalisation in Thailand.^33,52,92,105^ Papers by Thow et al^51^ and FAO et al,^58^ also indicated that measures often faced less objection in jurisdictions with a precedent for taxing harmful commodities.

####  International/Exogenous Factors

 The literature demonstrated that international factors had important and diverse influences on policy processes. Global factors supporting and inhibiting fiscal measures were identified in 51 papers (68%) and linked to trade agreements, the influence of multinational corporations and cross-border policy harmonisation.

 The imposition of trade agreements on the control of harmful commodities was discussed in several papers. As outlined by Ferreira-Borges et al,^89^ trade agreements designed to reduce trade barriers, promote competition, lower prices and encourage consumption generally sit at odds with the goals of fiscal measures. By constraining regulatory action, trade agreements can limit policy space and fuel inter-ministerial conflict between trade and health.^15,24,33,48,50,52,59,62,73,89^ By provoking disputes or sanctions and impeding access to bodies such as the World Trade Organization (WTO), trade conditions complicate the design of fiscal measures.^50,52,59^ Further, as argued by Baker et al,^53^ past trade litigation is often a barrier to future measures in different jurisdictions, a condition known as “regulatory chill.” LMICs are particularly vulnerable to the threat of retaliative action given their more constrained trade, economic and legal capacities.^53^

 The provision of foreign aid based on particular trade conditions determined by donors can also hamper the implementation of fiscal measures in LMICs. New Zealand’s threat of sanctions against a number of Pacific island nations for their health-related bans and proposed bans on turkey tails and mutton flaps exemplified this.^50,62^ Bump and Reich^33^ assert that such action demonstrates the power afforded to industry in influencing countries and, by extension, global trade policy. However, despite the limitations imposed by trade agreements, there is growing potential for evidence-based carve-outs, permitting discriminatory taxes on products proven to be detrimental to health.^24,50^ Moreover, the literature also highlighted WTO accession mandates and the threat of sanctions to have instigated government-wide review of fiscal measures. Fuelled by a sense of urgency, sanctions and accession parameters favoured multisectoral-supported fiscal measures in Thailand, the Philippines and Samoa.^33,48,52,62,92,105^

 The growing transnational nature of industries themselves is also responsible for stalling and impeding the adoption of measures. Holden and Lee^100^ outline that the multinational nature of many harmful commodity producers fosters transnational coordination. Transnational action by corporations commonly includes global counter-advertisement and lobbying,^15,66,98,100^ the development of transnational front groups,^57,60,77,93,103^ cross-jurisdictional information sharing,^100^ and offshoring or restructuring to minimise loss of market share or profitability.^86,95,100,107^ The transnational nature of negative retaliative action was most apparent in contexts where measures were not unanimously supported by domestic actors; reigniting inter-ministerial conflicts between trade and health.^33,73^

 Regional and global coalition building and direct support provision from multilateral organisations has however been advantageous in countering multinational corporations and supporting fiscal measures in LMICs. Information sharing and lesson-drawing commonly influenced policy processes and promoted policy transfer.^42,45,48,52,54,61,71,78,100,103^ Examples include the establishment of health promotion foundations, following successes of the Australian VicHealth model,^15,42-52^ and regional harmonisation of taxation to mitigate smuggling.^48,54,61,78,100,103^ Hoe et al^109^ also emphasised cross-country comparisons to be advantageous in informing policy processes, elevating dormant problems and their potential solutions onto national and international policy agendas.

 Similarly, policies and objectives that have achieved global consensus, such as FCTC and the SDGs, also support the design and implementation of fiscal measures. By fuelling international political momentum which, Bump and Reich argue,^33^ has the potential to trump domestic affairs, dominant global health discourse provides a prominent frame for issue conceptualisation and timely state compliance acts as a signifier of responsive governance. For example, Tobacco-related papers consistently identified FCTC as instrumental in issue identification and the design of state responses.^42,65,68,72,74,78,79,82,85,87,97,103,105,108-110^ As outlined by Tam et al^103^ and others, FCTC’s legally binding tobacco taxation provisions have fostered a sustained commitment to effective fiscal measures amongst signatory nations.^79,85,103,108,109^ Further, despite Indonesia not being a signatory, Achadi et al^65^ and Rosser^68^ also identify FCTC to have garnered momentum and supported a review of tobacco control measures in light of the framework’s health and economic provisions. While not legally binding, a range of other global policies, including the NCD Best Buys and global and regional action plans on NCDs have also aided consensus building and mainstreamed fiscal solutions.^15,76,80,87^ More lateral global health objectives, including the SDGs and universal health coverage, have also been leveraged by states to justify fiscal measures as a means of increasing health budgets.^46,48,58,63^

 Drawing on detail included in Supplementary file 4 and narrative summaries provided above, a summary of the state of the literature on health fiscal measures literature can be found in Table 2.

**Table 2:** State of Fiscal Measures Evidence Delineated by Subject Matter, Methodology/Focus and Content of Analysis

**Subject**	There was a concentration of papers analysing tobacco-specific fiscal measures and an under-representation of research focused on food, alcohol or other beverage-related fiscal measures.
**Methodology/focus**	Majority of the included literature is less than a decade old pointing to the relative infancy of this area of policy analysis. The literature demonstrated a concentration of papers analysing policy actors and, in particular, the influence of industry on the design and implementation of measures. Insufficient attention was paid to the influence of neoliberalism and power dynamics on the policy process.
**Content of analysis **	Local evidence, policy championing by political elites, inter-ministerial support and engagement and favourable global winds for change were identified as drivers of fiscal measures. Industry influence and retaliation, trade insecurity and regulatory chill, inter-ministerial policy disharmony and vested interests were identified to have challenged or prevented the design and implementation of fiscal measures.

## Discussion

 The design and implementation of health-related fiscal measures in LMICs is shaped by a complex network of factors that vary across contexts. Nonetheless some commonalities were identified by this scoping review. Fiscal measures were more likely to be implemented when diverse local health and economic evidence sources were available; policies were championed by those in government and had inter-ministerial support; stakeholders from different sectors engaged in regular, open dialogue; and when regional and global political winds favoured change. Fiscal measures were less likely to be adopted when framing of issues and solutions were influenced by industry; real and perceived retaliative threats were made by powerful actors; during political climates of trade insecurity and regulatory chill; and when disharmony and vested interests prevented policy consensus within government. Overall, these factors suggest some important distinctions between HICs and LMICs in the design and implementation of fiscal measures, as is summarised in Table 3.

**Table 3:** A Summary Comparison of Study Findings With Recent HIC Literature^22,24,28,42,128^

	**Factors Similar Across LMICs and HICs**	**Factors Unique to LMICs**
Content	A common desire to balance the benefits of earmarking with the risk such mechanism poses to governance of public sector financing. Variance in taxation rates and scope across countries and between different products.	
Actors	Industry influence commonly stalls and mitigates fiscal measures. Policy championing by state actors often accelerates implementation.	Industry influence is more pervasive, and nations experience constrained capacities to enforce industry-relevant regulations. Greater role for multilateral financial and technical input into the design and implementation of measures.
Process	Limited research on the policy process of fiscal measures. The important influence of the framing of policy objectives on public and political debate. Inter-ministerial support for measures commonly accelerate their adoption. Embedding fiscal health measures in broader fiscal reform garners inter-ministerial support and can accelerate adoption.	Limited context-specific evidence and constrained capacities to undertake policy-relevant research. More acute inter-ministerial power asymmetries. More constrained capacities for implementing and evaluating measures.
Context	Historic precedence for taxing commodities has benefits from current-day advocates who support such measures.	Constrained national budgets can incentivise revenue generation through fiscal measures but limit resources made available for effective design and implementation. A higher prevalence of communicable diseases and lower health budgets constrain NCD-related action.

Abbreviations: LMICs, low- and middle-income countries, HICs, high-income countries; NCDs, non-communicable diseases.

###  Gaps in the Analysis Food, Alcohol and Other Beverages 

 This review demonstrated a substantial gap in research focused on food, alcohol or other beverage-specific fiscal measures, with the current literature reflective of the sustained global emphasis on tobacco regulation and taxation. Given the increasing momentum for broader policies in response to NCDs, the lack of in-depth analysis devoted to these areas signifies a failure to provide relevant and actionable evidence to support the design and implementation of fiscal measures. While lesson-drawing from tobacco is valid and, as emphasised by Dorfman et al,^129^ Nguyen et al,^130^ and Brownell and Warner,^131^ other industries have followed tactics used to resist tobacco regulation, an over-reliance on tobacco-specific literature fails to acknowledge the differences associated with other harmful commodities. There is hence a need for greater depth of research focused on a range of harmful commodity-related fiscal measures to overcome the unstated assumption implied by much of the literature: that mechanisms used in tobacco taxation are directly replicable to measures targeting foods, alcohol and other beverages. For example, the literature’s predominant representation of industry as a homogenous entity risks over-simplifying policy challenges and may negate the influence of consumer perception and the versatility of the food and beverage industry in shaping regulations.

###  Recognising Government and Civil Society Alongside Industry Actors 

 This review also highlighted gaps in the breadth of policy actors identified, with many papers focused solely on the influence of industry. While important, particularly given the immense power afforded to industry actors in the political economies of LMICs, the literature’s preoccupation with industry precludes a more comprehensive assessment of the network of actors who influence the design and implementation of fiscal measures. For example, although only 38 studies (51%) examined inter-ministerial dynamics, sound relationships and agreement between state actors was pivotal to the fiscal measures’ success, and inter-ministerial conflicts often stalled or prevented measures. Given that public policy ultimately constitutes decisions made by state actors, the lack of attention to process dynamics and the views and actions of public persons and bodies is a critical gap in our understanding of how state actors negotiate competing interests.

###  The Need to Explore Root Causes

 Despite the emphasis placed on the influence of industry across the literature, very few papers explored potential root causes, with just Mambulu et al^98^ identifying neoliberalism as an underpinning determinant of private sector power. Global and local ideologies and dominant discourse are important contextual factors influencing the drive for and design of fiscal measures.^132,133^ With neoliberal policy preferences explicitly exported to LMICs as loan conditions from the IMF and World Bank in the 1990s,^132^ the pursuit of freer markets, with the promise of greater personal freedoms, peace and prosperity, remains a dominant global discourse. Encouraging trade, discouraging market regulation and, in turn, arguably promoting ‘consumptagenic systems,’^134^ neoliberalism sits at the heart of the escalating burden of NCDs and dictates the terms of potential remedial action.^135^ The scale, reach and wealth of corporations, particularly multi-national conglomerates, and their ability to mitigate and manipulate fiscal measures highlights an important power asymmetry between countries’ health interests and the economic interests of powerful private actors. However, some promise is found in recent support for fiscal measures by the IMF and World Bank and the implementation of measures by conservative governments.^24,60,126^ The altered stance of these agencies and governments demonstrates the possibility of reorientating the neoliberal agenda to recognise the importance and potential returns associated with investing in the health of ‘human capital.’^136^ Further research is necessary to explore the breadth of neoliberalism’s influence, as we currently have an incomplete picture of the barriers, tensions and political opportunities in assessing the feasibility of fiscal measures as a form of responsive public policy.

###  Recognising Other Forms of Evidence

 Many studies discussed the need for and use of health and economic evidence in the enactment of fiscal measures. However, while plentiful, evidence was almost exclusively discussed in relation to agenda setting. For example, country-specific data on disease prevalence and consumption of harmful commodities were identified as integral in driving public and political momentum for change. Yet analyses of policy design processes and policy content often demonstrated that technical detail became obsolete in negotiation processes, when garnering support from diverse stakeholders was paramount. This demonstrates a disjuncture in the evidence used during agenda setting and that used in the design of fiscal measures. Further, echoing Bump, Reich, Chantornvong and others,^33,73,92,105,137^ a deepening of policy analysis and recognition of other types of evidence necessary to inform the design and implementation of public policy is required. For example, integration of political economy considerations into prospective and retrospective analysis is likely to bring to light important dynamics integral to successful policy.

## Strengths and Limitations

 This study draws inspiration from the political economy approach outlined in Bump and Reich’s seminal work,^33^ but is distinct in its inclusion of fiscal measures relating to a broad range of harmful commodities (not just tobacco-related products). Our focus on LMICs also differentiates this analysis from more globally oriented work by Wright et al^25^ and Hagenaar et al.^24^ Application of a systematic approach and theory-driven analysis also allow us to add depth to observations made in Bridge and colleagues’ more general exploration of LMIC experiences and potential pitfalls in sugar-sweetened beverage taxation.^34^

 Several limitations must also be noted. The choice of databases, repositories and exclusion of primary policy sources may have inadvertently limited the identification of relevant papers. Given the breadth of languages spoken across LMICs, the exclusion of papers published in languages other than English may also have led to the exclusion of relevant articles. While developed iteratively, predominately single author screening, coding and analysis may also have unintentionally excluded information of relevance although robust discussion between authors and review by the third author was designed to minimise this. The study’s reliance on secondary sources also creates the potential for misinterpretation. Embedding expert consultation as a final stage in the study’s design may also have strengthened findings.

## Conclusion

 This study’s identification and mapping of literature exploring and explaining the policy process of fiscal measures in LMICs reveals important findings for policy-makers and researchers alike.

 Highlighting critical and interconnected factors influencing fiscal measures in LMICs, this study identifies a number of valuable lessons for future fiscal measures. The use of local health and economic evidence, policy championing, multisectoral engagement and inter-ministerial support, and global or regional momentum and technical support appear beneficial to the design and implementation of measures. By contrast industry framing and potential retaliation, vested interests and policy disjuncture across government were common factors associated with stalled or mitigated measures. Recognition of the presence or absence of these factors and intentional planning with such considerations in mind may hence support LMIC policy-makers in designing and implementing effective fiscal measures.

 While acknowledging that what is known should inform policy, it is also pivotal that what remains unknown informs future research. As such, this study has highlighted considerable gaps in our understanding of the global, regional and national political economies which shape fiscal measures. A greater emphasis on empirical research that seeks to understand the context-specific power dynamics and the political intricacies of processes associated with the design and implementation of fiscal measures in LMICs is hence integral.

 The growing burden of NCDs, and how it manifests in LMICs, is itself a product of political and economic forces. Effective policies responses hence demand evidence that acknowledge and account for such political economies.

## Ethical issues

 Not applicable.

## Competing interests

 Authors declare that they have no competing interests.

## Authors’ contributions

 LME and SMT conceived of the review. Data collection, charting, mapping, interpretation and analysis tasks were undertaken by LME with guidance from SMT and SLD. LME wrote the first draft of the manuscript and SMT and SLD provided critical input and feedback.

## Authors’ affiliations

^1^College of Public Health, Medical and Veterinary Sciences, James Cook University, Townsville, QLD, Australia. ^2^School of Public Health & Social Work, Queensland University of Technology, Brisbane, QLD, Australia. ^3^Department of International Health, Johns Hopkins School of Public Health, Baltimore, MD, USA. ^4^Institute for Global Health, University College London, London, UK. ^5^Nossal Institute for Global Health, University of Melbourne, Melbourne, Australia.

## Supplementary files


Supplementary file 1. Search Strategy.

Supplementary file 2. Criteria for Inclusion and Exclusion.

Supplementary file 3. Initial Codebook.

Supplementary file 4. Included Papers’ Alignment With the Domains of the Walt and Gilson Policy Triangle.
