# Supplementary material for: Health Taxes on Tobacco, Alcohol, Food and Drinks in Low- and Middle-Income Countries: A Scoping Review of Policy Content, Actors, Process and Context
Source: Int J Health Policy Manag. 2020 Sep 6;11(4):414–28. doi: 10.34172/ijhpm.2020.170 (PMC9309941; doi:10.34172/ijhpm.2020.170)
Supplement: Supplementary file 1 — Search Strategy. [file ijhpm-11-414-s001.pdf]

Supplementary file 1. Search Strategy

Health policy - “health poli*” OR “policy implementation” OR “policy analy*” OR “health agenda” OR “policy actor*” OR “political agenda” OR “political econom*” OR “political analys*” OR “policy develop*” OR “policy process*” OR “policy interest*” OR “policy discourse” OR “health advoca*” OR “health construct*” OR “policy influence*” OR “political influence” OR “political cycle” OR “public policy” OR “policy drivers” industry* OR “industry interference*” OR “industry influence*” OR “commercial interest*” OR “commercial influence*” OR “public interest*” OR “public opinion” OR “corporate behaviour” OR “corporate interest*” OR “corporate influence*” OR “private sector interest*” OR “private sector influence*” OR “private sector engagement” OR “stakeholder engag*” OR “stakeholder map*” OR “lobby*” OR “policy advoc*” OR “issue fram*” OR refram* OR “political priorit*” OR “policy transfer” OR “policy diffusion” OR “multisectoral polic*” OR “donor interest*” OR “donors interest*” OR “donor influence*” OR “donors influence” OR “health policy mak*” OR “health govern*” OR power OR “policy instrument*” OR “policy cohesion” OR “policy measure*”
Regulatory – “fiscal” OR “regulat*” OR tax* OR law* OR legislat* OR “behaviour control” OR “behaviour change” OR “behavioural change” OR incentiv* OR disincentiv* OR “social control*” OR “trade agreement*” OR “trade polic*”
LMICs – Individual LMIC countries as per World Bank[41] 2019 Country Grouping OR LMIC continents & sub-continents OR "developing countr*" OR "lower income countr*" "low and middle income countr*" OR “low income econom*” OR “low income countr*” OR “lower-middle income countr*” OR “lower-middle-income econom*” OR "developing econom*" OR "transitional countr*" OR “transitional econom*”
NCDs – "social determinant*" OR "chronic disease*" OR “chronic illness*” OR "noncommunicable disease*" OR "non communicable disease*" OR "non-communicable disease*" OR NCD* OR "risk factor*" OR cancer* OR diabet* OR “chronic obstructive pulmonary disease” OR emphysema OR “cardiovascular disease*” OR hypertensi* OR malnutrition OR overnutrition OR "over-nutrition" OR obesity OR overweight OR obesogenic OR unhealthy OR nutrition OR “lifestyle disease*” OR “lifestyle-related”
Commodities - tobacco OR smok* OR cigarette* OR soda OR “soft drink*” OR “softdrink*” OR “sugar sweetened beverage*” OR “sugar-sweetened beverage*” OR “carbonated beverage*” OR sugar* OR “alcohol” OR “alco-pop” OR “sin” OR “junk food*” OR “high salt” OR “calorie-dense” OR “salt reduc*” OR “high fat*” OR “saturated fat” OR “energy dense” OR “unhealthy commodit*” OR “harmful corporat*” OR “ultra processed food*” OR “trans-fat*” OR “trans fat*” OR diet* OR “lifestyle-related” OR “commercial determinant*” OR “food industr*”

Search Results: Date of Search: March 21, 2019

Database	Search String	Records Obtained
Embase	Health policy AND Regulatory AND	645
PubMed		180
ProQuest		2687

Limits: NOFT, English, Trade journals/gov & official publications/ books/ scholarly journals/ dissertations & theses only.	LMICs AND NCDs OR Commodities	
Scopus<br>Limits: Excluded engineering, energy, biochemistry, computer science, immunology, mathematics, chemical engineering, chemistry, veterinary & physics subject areas		1535
