# Supplementary material for: Health Taxes on Tobacco, Alcohol, Food and Drinks in Low- and Middle-Income Countries: A Scoping Review of Policy Content, Actors, Process and Context
Source: Int J Health Policy Manag. 2020 Sep 6;11(4):414–28. doi: 10.34172/ijhpm.2020.170 (PMC9309941; doi:10.34172/ijhpm.2020.170)
Supplement: Supplementary file 2 — Criteria for Inclusion and Exclusion. [file ijhpm-11-414-s002.pdf]

Supplementary file 2. Criteria for Inclusion and Exclusion

Title & Abstract Screening

Inclusion:

- Published in English
- No time period of focus
- Academic & grey literature sources
- Provides detail on at least one LMIC
- Focuses on public policy process
- Articulates or explores links between fiscal measures and health (particularly NCDs, their risk factors or harmful commodities)

Exclusion:

- Prevalence studies
- Non-empirical, predictive or projection studies
- Experimental/intervention studies
- Health or economic focused outcome & impact evaluations
  Studies focused on biomedical or pharmaceutical policies

Full Text Screening

- Empirical work or drawing on a sound empirical base
  Provides an account of agenda setting, design or implementation of fiscal measures targeting or responding to NCDs or their determinants.
