# Supplementary material for: Health Taxes on Tobacco, Alcohol, Food and Drinks in Low- and Middle-Income Countries: A Scoping Review of Policy Content, Actors, Process and Context
Source: Int J Health Policy Manag. 2020 Sep 6;11(4):414–28. doi: 10.34172/ijhpm.2020.170 (PMC9309941; doi:10.34172/ijhpm.2020.170)
Supplement: Supplementary file 3 — Initial Codebook. [file ijhpm-11-414-s003.pdf]

Supplementary file 3. Initial Codebook

Initial Codes:

- Intended use of revenue
- Policy content
- Purported use of the measure
- Conflicts of interest/Vested interests
- Reasoning: Defence of policy
- Reasoning: Defence of retaliatory action
- Recognition of harm associated with harmful commodities
- Industry influence
- Policy prioritisation
- Multisectoral action
- Civil society engagement
- Inter-ministerial action
- Political commitment/Leadership/Political will
- Types and use of frames
- Implementation details
- Evidence: Lack of or use of
- Global & regional context
- Relevant history
- Capacity constraints
- Local context
