# Supplementary material for: Health Taxes on Tobacco, Alcohol, Food and Drinks in Low- and Middle-Income Countries: A Scoping Review of Policy Content, Actors, Process and Context
Source: Int J Health Policy Manag. 2020 Sep 6;11(4):414–28. doi: 10.34172/ijhpm.2020.170 (PMC9309941; doi:10.34172/ijhpm.2020.170)
Supplement: Supplementary file 4 — Included Papers’ Alignment With the Domains of the Walt and Gilson Policy Triangle. [file ijhpm-11-414-s004.pdf]

Supplementary file 4. Included Papers' Alignment With the Domains of the Walt And Gilson Policy Triangle

Papers	Commodity	Economic Status	Region	Content		Actors				Process				Context			
				Earmarking	Tax Structure	Industry Influence	Policy Champions	Civil Society	Multilaterals	Framing	Evidence	Inter-ministerial dynamics	Implementation	Situational	Structural	Cultural	International
Abedian, van der Merwe, Wilkins & Jha, 1998	Tobacco	Upper Middle	Africa	x	x	x	x					x		x	x	x	x
Achadi, Soerojo & Barber, 2005	Tobacco	Lower Middle	Asia	x	x	x	x			x	x		x	x		x	x
Adebiyi & Popoola, 2018	Tobacco	Lower Middle	Africa			x				x		x			x		
Akitoby, Honda, Miyamoto, Primus & Sy, 2019	Combined	Low	Global		x									x			
Alechnowicz & Chapman, 2004	Tobacco	Lower Middle	Asia		x	x					x			x	x	x	
Asare, 2009	Tobacco	Upper Middle	Africa			x	x	x	x	x	x	x	x		x	x	x
Baker, Jones & Thow, 2017	Soft Drink	Multi-Region	Global	x		x		x	x	x	x	x		x	x		x
Barber & Ahsan, 2009	Tobacco	Lower Middle	Asia			x				x			x	x	x		
Barbieri, Sarmiento, Diaz del Castillo et al., 2014	Combined	Multi-Region	South & Central America				x	x				x		x			x
Barraclough & Morrow, 2010	Tobacco	Multi-Region	Asia			x	x	x				x		x	x	x	x
Bonilla-Chacín, Iglesias, Suaya, Trezzam & Macías, 2016	Combined	Upper Middle	South & Central America	x	x	x	x	x	x	x	x	x		x			x
Bump, Reich, Adeyi & Khetrapal, 2009	Tobacco	Multi-Region	Global			x		x	x	x	x	x	x	x	x		x

Bump & Reich, 2013	Tobacco	Multi-Region	Global			x		x		x	x	x			x	x	x
Cashin, Sparkes & Bloom, 2017	Combined	Multi-Region	Global	x						x		x			x		x
Casswell, 2013	Alcohol	Multi-Region	Global			x				x							
Chantornvong & McCargo, 2001	Tobacco	Upper Middle	Asia			x	x	x		x			x	x	x	x	x
Chantornvong, Collin, Dodgson et al., 2000	Tobacco	Multi-Region	Global			x	x			x	x			x	x	x	x
Charoenca, Kungskulniti, Mock, Hamann & Vathesatogkit, 2015	Combined	Upper Middle	Asia							x			x	x	x		x
Charoenca, Mock, Kungskulniti, Preechawong, Kojetin & Hamann, 2012	Tobacco	Upper Middle	Asia		x	x	x	x		x	x		x	x	x		x
Chavez, 2015.	Tobacco	Multi-Region	Asia	x		x						x			x		
Coriakula, Moodie, Waqa, Latu, Snowdon & Bell, 2018	Food	Upper Middle	Pacific	x	x	x	x	x			x	x	x	x			
Crosbie, Sebrié & Glantz, 2012	Tobacco	Upper Middle	South & Central America		x	x	x	x			x	x		x	x		x
Crosbie, Sosa, Glantz, 2016	Tobacco	Upper Middle	South & Central America	x	x	x		x	x		x		x		x		x
Crosbie, Sosa & Glantz, 2017	Tobacco	Upper Middle	South & Central America	x		x	x		x		x		x		x		
Drope, Chavez, Lencucha & McGrady, 2014	Tobacco	Lower Middle	Asia	x	x	x	x			x				x	x		x
Ferreira-Borges, Parry & Babor, 2017	Alcohol	Multi-Region	Africa			x				x	x	x		x	x	x	x
FAO & The Fiji National University C-POND, 2017	Food	Upper Middle	Pacific		x		x	x				x		x	x	x	x
Foster, Thow, Unwin, Alvarado & Samuels, 2018	Combined	Multi-Region	Global		x	x	x						x	x	x		x
García, Uribe & Iunes, 2017	Combined	Upper Middle	South & Central America			x		x			x	x		x			x
Gilmore, Fooks, Drope, Bialous & Jackson, 2015	Tobacco	Multi-Region	Global			x				x			x	x	x		

Gilmore, Collin & Townsend, 2007	Tobacco	Lower Middle	Middle East			x				x				x	x	x	x
Gilmore, 2012	Tobacco	Multi-Region	Global			x											
Hagenaars, Jeurissen & Klazinga, 2017	Combined	Multi-Region	Global	x	x	x				x	x		x	x	x		x
Hamann, Mock, Hense, Charoenca & Kungskulniti, 2012	Tobacco	Upper Middle	Asia	x				x	x		x						x
Higashi, Khuong, Ngo & Hill, 2011	Tobacco	Lower Middle	Asia			x	x	x	x	x	x	x	x	x	x		x
Higashi, Khuong, Ngo & Hill, 2013	Tobacco	Lower Middle	Asia	x		x				x	x	x		x	x		
Higashi, Ngo & Hill, 2012	Tobacco	Lower Middle	Asia			x	x			x	x	x		x	x		x
Hoe, Rodriguez, Üzümcüoğlu & Hydee, 2016	Tobacco	Upper Middle	Europe				x	x			x			x		x	x
Holden & Lee, 2011	Tobacco	Multi-Region	South & Central America			x	x							x	x		x
Hu, Lee & Mao, 2016	Tobacco	Upper Middle	Asia			x	x								x		
Iglesias, 2016.	Tobacco	Upper Middle	South & Central America	x	x	x				x	x		x				
Juma, Mapa-tassou, Mohamed et al., 2018	Combined	Multi-Region	Africa			x	x	x			x	x	x		x		x
Juma, Mohamed, Matanje Mwagomba et al., 2018	Combined	Multi-Region	Africa			x	x	x			x	x	x	x	x		x
Kaiser, Bredenkamp & Iglesias, 2016	Combined	Lower Middle	Asia	x	x	x	x	x		x	x	x		x	x	x	x
Krasovsky, 2010	Tobacco	Lower Middle	Europe			x	x										
MacKenzie & Collin, 2017	Tobacco	Lower Middle	Asia			x				x					x		
Malan & Leaver, 2003	Tobacco	Upper Middle	Africa			x	x	x		x	x	x	x	x	x	x	x
Mambulu, Mkandawire, Dixon, Mason, Rishworth & Luginaah, 2015	Alcohol	Low	Africa		x	x	x		x	x			x	x	x	x	x

Mapa-Tassou, Bonono, Assah et al., 2018	Tobacco	Lower Middle	Africa			x	x	x		x				x	x	x	x
Mialon, Swinburn, Wate, Tukana & Sacks, 2016	Food	Upper Middle	Pacific			x				x	x			x	x		x
Mohamed, Juma, Asiki & Kyobutungi, 2018	Tobacco	Lower Middle	Africa			x	x				x	x	x	x	x	x	x
Oladepo, Oluwasanu & Abiona, 2018	Tobacco	Lower Middle	Africa			x	x	x		x		x	x	x	x		x
Onagan, Ho & Chua, 2019	Soft Drink	Lower Middle	Asia		x	x	x		x	x	x	x	x	x			x
Owusu-Dabo, McNeill, Lewis, Gilmore & Britton, 2010	Tobacco	Lower Middle	Africa				x			x					x		
Parry, 2010	Alcohol	Upper Middle	Africa	x		x	x				x	x		x			x
Perez-Escamilla, Lutter, Rabadan-Diehl et al., 2017	Soft Drink	Multi-Region	South & Central America			x	x	x		x	x	x	x				x
Pitso & Obot, 2011	Alcohol	Upper Middle	Africa	x		x	x				x	x			x	x	
Ross, Tesche & Vellios, 2017	Tobacco	Multi-Region	Global			x											
Rosser, 2015	Tobacco	Lower Middle	Asia		x	x	x	x	x	x				x	x	x	x
Roubal, 2017	Soft Drink	Upper Middle	Africa	x	x	x		x	x	x	x	x			x		x
Samet, Wipfli, Perez-Padilla & Yach, 2006	Tobacco	Upper Middle	South & Central America	x		x											
Sanni, Hongoro, Ndinda & Wisdom, 2018	Tobacco	Multi-Region	Africa			x	x	x			x	x	x	x	x	x	x
Smith, Savell & Gilmore, 2013	Tobacco	Multi-Region	Global	x		x						x					
Snowdon & Thow, 2013	Combined	Multi-Region	Pacific	x	x	x	x			x	x		x	x	x	x	x
Tam J & van Walbeek, 2014	Tobacco	Upper Middle	Africa			x	x	x			x		x	x	x	x	x
Thow, Quested, Juventin, Kun, Khan & Swinburn, 2011	Soft Drink	Multi-Region	Pacific	x	x	x		x		x	x	x	x	x		x	

Thow, Reeve, Naseri, Martynd & Bollars, 2017	Food	Upper Middle	Pacific		x		x		x		x			x	x		x
Van Walbeek, 2003	Tobacco	Upper Middle	Africa	x		x	x	x		x	x	x		x	x	x	
Van Walbeek, 2004	Tobacco	Upper Middle	Africa			x	x	x		x	x		x	x	x	x	
Vateesatokit, 2003	Tobacco	Upper Middle	Asia	x	x	x	x	x		x	x	x				x	x
Walls, Liverani, Chheng & Parkhurst, 2017	Tobacco	Lower Middle	Asia			x	x			x	x		x		x	x	x
Williams, 2015	Soft Drink	Upper Middle	Asia		x	x				x					x	x	x
Wisdom, Juma, Mwagomba et al., 2018	Tobacco	Multi-Region	Africa			x	x	x			x	x	x	x	x	x	x
World Health Organization, 2016	Tobacco	Multi-Region	Global	x	x	x	x	x		x	x	x	x	x	x	x	x
World Health Organization, 2016	Combined	Multi-Region	Global	x	x	x	x	x			x	x		x	x		x
